# Advanced Solid-Phase Microextraction Techniques and Related Automation: A Review of Commercially Available Technologies

**DOI:** 10.1155/2022/8690569

**Published:** 2022-02-04

**Authors:** Stefano Dugheri, Nicola Mucci, Giovanni Cappelli, Lucia Trevisani, Alessandro Bonari, Elisabetta Bucaletti, Donato Squillaci, Giulio Arcangeli

**Affiliations:** ^1^Industrial Hygiene and Toxicology Laboratory, University Hospital Careggi, Florence, Italy; ^2^Department of Experimental and Clinical Medicine, University of Florence, Florence, Italy; ^3^General Laboratory, Careggi University Hospital, Florence, Italy

## Abstract

The solid-phase microextraction (SPME), invented by Pawliszyn in 1989, today has a renewed and growing use and interest in the scientific community with fourteen techniques currently available on the market. The miniaturization of traditional sample preparation devices fulfills the new request of an environmental friendly analytical chemistry. The recent upswing of these solid-phase microextraction technologies has brought new availability and range of robotic automation. The microextraction solutions propose today on the market can cover a wide variety of analytical fields and applications. This review reports on the state-of-the-art innovative solid-phase microextraction techniques, especially those used for chromatographic separation and mass-spectrometric detection, given the recent improvements in availability and range of automation techniques. The progressively implemented solid-phase microextraction techniques and related automated commercially available devices are classified and described to offer a valuable tool to summarize their potential combinations to face all the laboratories requirements in terms of analytical applications, robustness, sensitivity, and throughput.

## 1. Introduction

**Figure 1:**
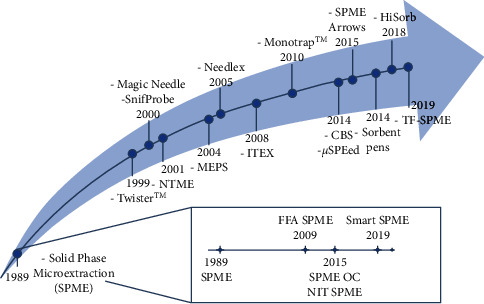
Timeline of the principal solid-phase microextraction techniques developed in recent years (SPME: solid-phase microextraction; FFA: fast fit assemblies; OC: overcoated; NIT: nitinol-core; NTME: needle trap microextraction; MEPS: microextraction by packed sorbent; ITEX: in-tube extraction; CBS: coated blade spray; TF: thin film).

The first and the most crucial step of any analytical procedure is the sample pretreatment. Furthermore, it is the bottleneck of an analytical process in gas chromatography (GC) and liquid chromatography (LC) applications [1]. When performing samples pretreatment, the principal reasons for extracting are obtaining a more purified sample, eliminating interfering compounds, and improving sensitivity for specific analytes. To adapt extraction methods already existing and to propose new approaches to save time, labor, and materials, significant endeavors have been made. The separation of the analytes from the sample matrix and their preconcentration are essential parts of the extraction procedure. The most well-known, broadly used, and generally accepted exhaustive extraction methods are liquid-liquid extraction (LLE) and solid-phase extraction (SPE); they provide easy quantification and highest sensitivity since all target compounds are separated from the sample [2, 3]. Nevertheless, rising environmental attention leads to friendly and greener technologies, resulting in the miniaturization of sample treatment methods. In the past 30 years, numerous miniaturized sample preparation techniques have been invented to displace LLE and SPE, taking miniaturized sample preparation devices advantages, namely, high speed, simultaneous sample concentration, automation, and possibility for direct injection of all analytes into the analytical instrument with a reduced amount or even without organic solvent [4–7]. Miniaturized methods are usually defined as nonexhaustive sample preparation techniques, requiring minimal extracting phase volume compared to the amount of the sample. The International Unit of Pure and Applied Chemistry (IUPAC) defined the MicroExtraction Techniques (METs) as those using a substantially smaller extraction phase than the sample volume [8]. The Solid-Phase MicroExtraction (SPME) in the coated fiber format is the first and most successful miniaturized sample preparation technique, which Pawliszyn invented in the early 1990s [9]. Some miniaturized techniques are designed and applied like exhaustive ones, such as In-Tube MicroExtraction (ITME) or Solid-Phase Dynamic Extraction (SPDE), with their drawbacks and advantages [1]. In the last decade, miniaturization has been more and more applied to chromatographic systems, saving both costs and time by automating the sampling procedures. Specifically, new techniques can simultaneously perform sampling collection, extraction, concentration, and introduction of the sample in the analytical system (Figure 1).

The substitution of conventional analytical techniques with miniaturized solutions in the latest years is noteworthy and is entirely consistent with the needs of Green Analytical Chemistry (GAC) [10], based on making the smart mixture of low-cost and environment-friendly methodologies. Consequently, in addition to the different miniaturized techniques, green solvents, as soon as the use of enhancer of the efficiency of sample preparation (i.e., high temperature and/or pressure, microwave and UV radiation, and ultrasound energy), are deployed in new analytical solutions and are extremely recommended [11]. Thus, the development of methods and tools, like eco-scale to evaluate the greenness of analytical procedure [12–16], as well as modelling chromatographic separations systems [17–19] and design of experiments software [20], has become essential. Concurrently, implementing greenness and complete automation in analytical processes has proliferated, increasing the usage of miniaturized techniques. This proliferation has produced savings on solvents, faster sample preparation, diminished costs and errors per analysis, improved traceability, and full instrument automation [21]. Even though the majority of chromatography laboratories already utilize autosamplers, this advanced instrumentation enables automation beyond just the injection. Several tools are on the market, comprising barcode reader, automatic change liners and syringes, decapper, heaters, stirrers, shaking and/or filtration modules, solvent addition, and Local Area Network (LAN) connections, expanding applicability and efficiency [22].

In June 2021, to provide a comprehensive view of the latest METs and their related automation systems, a research for scientific sources in bibliographic databases of peer-reviewed journals (PubMed, Web of Science) has been conducted. In this perspective, main sources from 2000 have been considered. This first research has been successively integrated from Google, Google Scholar, and Google Patent with nonscientific sources, like application notes and manufacturer datasheets, available on manufacturers or supplier websites.

In this study, the METs are classified according to their geometry and their characteristic of being exhaustive or nonexhaustive. Conversely, previous studies classified them generally into two categories according to Jochmann et al. [23] and Nerín et al. [24]: coated, as SPME fiber or in-needle/in ITME methods, and tubes or needles filled with sorbent material. The aim of this review is to show the fourteen commercially available solid-phase METs used for chromatographic separation and mass-spectrometric detection with their main features, highlighting the latest upswing in the availability and range of their automation.

## 2. Review

In analytical chemistry, sample preparation occupies 70–80% of the time process [25]. This drawback boosted the development of solutions to minimize the time, and the work requested and, moreover, to face the increasing demand for more sensitive analytical methods [26]. METs meet these needs by coupling sampling and sample preparation steps into one, decreasing the time of analysis and enhancing the accuracy. The volume and kind of sorbent phase, the geometry of the devices, and the extraction efficiency towards targeted analytes could affect the analytical method in terms of sensitivity and capability. One primary consideration about METs is their exhaustive or nonexhaustive nature.

### 2.1. Microextraction Theory

The exhaustive microextraction process can be interpreted as frontal chromatography since the continually applied sample flow to the sorbent bed. In this scenario, the concentration (*C*(*x*, *t*)), for a short bed column or a coated capillary in the ITME, can be defined via the following equation:

(1) $$\begin{array}{c} C\left(x,t\right)=\frac{1}{2}\;C_{S}\left(1-\text{erf}\left(\frac{x-\left(u\;t/\left(L\;\left(1+k\right)\right)\right)}{\sigma \;L\;\sqrt{2}}\right)\right)-\frac{1}{2}\;C_{S}\times \exp\left(2\;n\right)\times \text{erf}\left(1-\text{erf}\left(\frac{x-\left(u\;t/\left(L\;\left(1+k\right)\right)\right)}{\sigma \;L\;\sqrt{2}}\right)\right). \end{array}$$

According to equation (1), the concentration of the analyte (*C*) extracted via a flow-through system (function of the variables of time *t* and position *x*) is governed by the initial concentration of the analyte in the medium (*C*_*S*_), the relative position (*x*) along the extracting phase, the linear velocity of the sample through the column (*u*), the time (*t*), the length (*L*) of the extracting phase, the retention factor (*k*), the root mean square dispersion of the front (*σ*), and the theoretical plate number (*n*) [27]. One of the main aspects of exhaustive techniques is the breakthrough that occurs when the sorbent bed is saturated by the analytes [28]. The breakthrough volume (bV) is the maximum amount of sample that can be loaded to an exhaustive phase without a considerable loss of the target compound. At first, the extractive sorbent phase quantitatively retained the analyte, till its retention capacity is overcome by the sample volume; after that, the addition of sample to the trap will not be retained by the sorbent, and ultimately, the entering and exiting concentration of analyte will be equivalent [29]. The quantity of bV for each analyte at the 1% (*V*_*b*_) is linked to the retention factor on the sorbent (*k*), theoretical plates number (*n*), and the void volume of the sample on the device (*V*_0_).

(2) $$\begin{array}{c} V_{b}=\left(1+k\right)\;\left(1-\frac{2.3}{\sqrt{n}}\right)\;V_{0}. \end{array}$$

Large bV and consequent sensitive ITME, the geometry, the sorbent material's physical-chemical properties, and sample type should be considered to obtain a high-volume flow rate. For example, a larger diameter of the trap with a longer sorbent bed can increase the capacity.

The concentration profiles in the classic exhaustive methods, as LLE or SPE, for individual analytes are a function of their affinity to the extraction phase, defined by the distribution coefficient, which, in turn, represents the retention in the system. In exhaustive MET, as an equilibrium-based scenario, the complete breakthrough is reached all the time, and the highest efficiency is acquired independently of experimental settings and volume of the sample, although, likewise to SPE, obstruction might affect capillary design and extra steps may be requested [30].

On the contrary, nonexhaustive MET, as SPME, partially extracts analytes by direct immersion or via headspace, relying on the partition equilibrium between the coating and the sample. In this condition, only a slight portion of the analytes is adsorbed/absorbed to the extraction phase, and subsequently, it can be completely desorbed into the GC or LC for analysis, enabling a sensitivity gain.

The quantitation of analytes extracted by nonexhaustive methods is extremely strict to the distribution of analytes between the sample and the coating, which is affected by temperature, agitation, ionic strength, pH, and matrix polarity of the sample [20]. Therefore, careful calibration and optimization are needed to develop a robust quantitative method. Since, in nonexhaustive methods, the constant of distribution and the amount of the extraction phase define the extracted quantity, the use of open-bed geometry represents the solution to overcome the cited limits of ITME methods. Exhaustive and nonexhaustive methods showed theoretically similar extraction recoveries, considering a scenario with sorbents of analogous features and quantities.

The distribution constant (*K*_*fs*_) in nonexhaustive MET relates with the retention factor (*k*) in ITME, by the following equation [30]:

(3) $$\begin{array}{c} k=K_{fs}\frac{V_{e}}{V_{0}}, \end{array}$$

where (*K*_*fs*_) is the distribution constant of extractive phase/sample matrix, (*V*_*e*_) is the volume of extractive phase in ITME device, and (*V*_0_) is the nonexhaustive MET device void volume [30]. The *K*_*fs*_ value for nonexhaustive device coating can be defined by the following equation:

(4) $$\begin{array}{c} n_{e}=\frac{K_{fs}\;V_{f}\;V_{s}\;C_{s}}{K_{fs}\;V_{f}+V_{s}}, \end{array}$$

where *n*_*e*_ is the quantity of analyte extracted at equilibrium, *C*_*s*_ is the initial concentration of a target compound in the sample, *V*_*s*_ is the sample volume, *V*_*f*_ is the coating volume, and *K*_*fs*_ is the distribution constant of the analyte between the sample matrix and the coating [30].

As indicated above, the exhaustive MET allows easy quantitation and high sensitivity, but the bV restricts the useable sample volume. When the affinity of the analytes differs considerably towards the coating, the limitation due to the bV for less retained substances might be an important restriction in the exhaustive method [31]. This limitation is reflected in lower enrichment of more retained analytes, affecting the efficiency of exhaustive extraction methods. Thus, the exhaustive nature of the ITME technologies (SPDE, ITEX, Sorbent pens, MEPS, *μ*SPE, SniffProbe, and Agilent probe) requires attention to ensure that, during the extraction phase, no breakthrough occurs. Concerning the nonexhaustive METs, there is no restriction of bV. Despite the smaller extraction phase volume, the quantity of more retained compounds extracted by these techniques could be higher than those resulting from exhaustive devices when target chemicals vary in polarity. The exhaustive techniques are much better for sensitivity, at least one magnitude better than nonexhaustive ones, but these techniques are less selective. Moreover, the lack of volume limit in nonexhaustive techniques allows determinations in living organisms or direct on-site sampling from the investigated systems [32, 33]. In effect, samplings from large systems utilizing tools like SPME or Thin Film-SPME (TF-SPME) result in slight depletion of analytes [34].

The MET devices are particularly appropriate for automatized sample treatment. Miniaturized devices that enable studies with a reduction in operation steps and analytical errors have been widely used [7]. Automation simplifies time-consuming sample preparation procedures and increases the accuracy of the analysis. Numerous commercially available robotized autosamplers have been produced to handle automated extraction/desorption via different microextraction supports in online or offline mode. The automation can interest a specific operation or the whole process, and it may operate on one sample or on several samples simultaneously. The automation process could be sequential (one sample, all the steps one by one), batch-based (each unit of the process is carried out for all the samples before the next unit is performed), concurrent sequential (more than one sample is in the chain of operations), and parallel batch-based (batch of samples are run in parallel). These last two settings represent the goal of new automatized sample preparation units because they allow the main gain in time of analysis and productivity. New solutions, coupling selective sorbents, METs, advanced controlling systems, and platforms, could provide complete and time-dependent information of compounds of interest.

Enhancements of high-throughput robotic microextraction systems have had an important impact on increasing the precision and throughput of analytical sessions and minimizing their time and cost. Moreover, the miniaturization of the extraction devices, coupled with the new portable, high-sensitive analyzers, and customized direct injection ports, could open new fields of applications, occupational, or forensic particularly. In 2019, Agilent Technologies (Santa Clara, US) introduced a new injection system, the QuickProbe, based on a vaporization inlet that is open to ambient air while having helium purged-flow protection to eliminate air leakage into the QuickProbe and MS ion source [35]. It is an innovative sample introduction technology that uses a thin glass tube as a sampling probe, touching directly liquid, solid, or powder samples before introducing it into the customized inlet for three to six seconds for vaporization. QuickProbe system could also provide rapid separation thanks to tailored QuickProbe column installed on Agilent analyzers [36].

### 2.2. Solid-Phase Microextraction Exhaustive and Nonexhaustive Techniques

The flexibility of geometry of SPME techniques opens chances to create better, modern, and greener solutions for sample preparation; thus, several exhaustive and nonexhaustive METs have been introduced [37] (Tables 1 and 2).

#### 2.2.1. Nonexhaustive Microextraction Techniques

The SPME technique was patented in 1989 [9], and Supelco (Bellefonte, US) introduced the first commercial SPME device in 1993 [38], which is improved in 2001 with a customized holder for sampling. Today, other companies propose SPME-like devices as Restek Corporation (Bellefonte, USA) or PAS Technologies (Magdala, Germany), which produces only polydimethylsiloxane (PDMS) fibers. The SPME is a fiber, contained in a stainless-steel needle coated with a liquid or solid sorbent phase. It was applied to sample various analytes from different matrixes, either gases or liquids [39]. Due to its geometry, one of its biggest drawbacks is fragility and lack of stability [40]. To face these features, Supelco has been studying on the development of SPME to obtain better physical stability for persistent reusability. Within this framework, GC-amenable StableFlex SPME fibers were proposed to improve the endurance of the fiber by coating, using the same extraction phases of traditional fused-silica core fibers, a more flexible fused-silica core. This extraction phase is in part connected to the core, leading to additional coating and fiber endurance, whilst maintaining the flexibility of the device. Lately, Supelco has released SPME fibers based on Nitinol-core (NiTi) SPME fiber, a metal alloy-based fiber made with a material characterized by high flexibility with better inertness compared to stainless steel [41]. This thinner metal alloy provides extra flexibility, whilst the needle is reinforced thanks to the thicker alloy in the plunger. Additionally, the tip is beveled to help the septa piercing of this thin needle; this kind of tip requires a septumless sealing systems to avoid septa coring.

Supelco overcoated SPME (OC) was proposed to address the challenges associated with immersion SPME: direct immersion in difficult matrixes could lead to sticking macromolecules to the adsorptive coating of SPME, causing a reduction in fiber lifetime [42]. When fiber is immersed in samples, the overcoating extends fiber life by 75–100% by reducing matrix build-up on fiber, increasing its durability, sealing ends, preventing matrix wicking, and slightly reducing fiber polarity, but increasing fiber capacity. Since their launching, the SPME fibers that have been developed have been adopted in various published applications, both as an active or passive sampler, presenting rewarding results [43–50].

To deal with two of the major issues of SPME, specifically, the fragility of the fiber and full automation of SPME-based processes, since 2009, have been introduced other devices in the market. In that year, Chromline (Prato, Italy), in collaboration with Supelco developed the SPME Fast Fit Fiber Assembly (FFA-SPME) that allows the exchange of SPME fibers completely automated thanks to a dedicated autosampler [51]. In 2016, a SPME syringe, called the Custodion, was released for the Guardion GC-TMS system by Torion Technologies Inc. (American Fork, US), as well as other GC and GC/MS systems; it included a memory chip that logs the syringe internal diameter (I.D.) and other programmed information (metadata) about the sample to increase data consistency and traceability [52]. Moreover, on the Guardion GC-TMS, the Custodion also triggers the injection and analysis to start without needing to push a “start analysis” button, allowing high-throughput full automated analytical session [53].

In 2019, Supelco Smart SPME fibers assemblies combined innovative SPME devices with Smart technology for seamless sample preparation. Each device is made by a SPME fiber inside a holder and a chip coded with fiber chemistry type, dimensions, lot number, number of fiber stroke count, injection and conditioning durations, usage dates, and expiration date. The compatibility of SPME and related tools on modern autosampler, as CTC PAL (CTC Analytics, Zwingen, Switzerland) or HTA (Brescia, Italy), allows the development of a more efficient analytical method and completely automated sample preparation, leading to increased productivity and traceability and a reduction of personnel related costs. Moreover, miniaturization and automatization are crucial in the development of methods in line with the GAC, and the SPME has proved to be one of the most applied and versatile techniques [54, 55].

Nowadays, other companies are working on SPME technologies using the same idea: Restek Corporation designed the PAL SPME Arrows in 2015 [56]. This device is a larger-diameter SPME probe with rugged construction that ensures longer life, higher sample throughput, and better sensitivity over traditional SPME fibers. It contains greater phase volume than normal SPME fibers, allowing the extraction of more target analytes. The SPME Arrows also had sorbent fibers into a stainless-steel cylinder with an internal rod and an outer sheath to safeguard the sorbent from mechanical damage and minimize the analyte loss [22].

In 2018, the Centri, a completely automated multimode platform to sample and concentrate for GC–MS, was released by Markes Int. Inc. (Sacramento, US) to automate sampling, solid-phase METs usage reliably as SPME, SPME trap, HiSorb-, and thermal desorption. This new platform automates the sampling process, preconcentration, and GC injection for liquids, solids, and vapors, allowing an improvement of productivity. One of the main features of this autosampler is the SPME-trap, compatible with conventional SPME and Arrow. This cryogen-free focusing trap allows multiple extractions from a single vial or from replicate samples in multiple vials onto the trap before desorption, increasing both the analyte response and the number of compounds identified [57, 58].

A different design was adopted in the nonexhaustive HiSorb Sorptive Extraction probes from Markes Int. Inc., which in 2016 launched these PDMS extraction probes. HiSorbs are a thin rod of inert material wrapped with a short sleeve of PDMS as a sorbent phase that can isolate analytes from gaseous and aqueous samples [57]. Using a relatively larger volume of PDMS sorbent than conventional SPME, fixed on a more robust metal probe, HiSorb obtains high sensitivity and robustness to matrix interferences, at the same time. Furthermore, these probes can be managed in full automation by Centri autosampler, which presents a customized injection port for their thermal desorption.

One of the oldest METs in terms of conception, the Stir Bar Sorptive Extraction (SBSE), patented in US in 2002 [59], was proposed by Baltussen in 1999 [60]. SBSE was a solventless MET considered like a variation of SPME. The device is a magnetic stir bar closed in a glass shell covered with a sorbent layer. The first sorbent applied was a PDMS layer with a thickness of 0.5–1 mm, with typical stirring times for equilibration between 30 and 60 min, depending on sample volume and the stirring speed, and detection limits in the low ng/L range for a wide selection of analytes. These stir bars are excellent enrichment devices for preconcentrating different compounds from aqueous samples [61, 62]. The PDMS sorbent generates suitable blanks, the coated stir bars show no deterioration after 100 extractions, and the amount of applied PDMS-coating outgrows SPME, so extremely low detection limits can be reached. PDMS coated stir bars are now commercially available, as Twister, from Gerstel GmbH (Mülheim an der Ruhr, Germany). These stirring rods are coated with a 1 mm layer of PDMS and are available in two sizes, 10 mm L × 3.2 mm o.d., used for 1–50 mL sample volumes, and 40 mm L × 3.2 mm o.d., used for 100–250 mL ones. In 2012, a new extraction phase was released by Gerstel GmbH, the ethylene glycol (EG) silicone sorbent phase that complements the PDMS phase, allowing sampling of a broader range of compounds [63]. Twicester is a peculiar application of Twister that allows the positioning via a magnet of one or more device on the inner wall of a vial for more efficient sample extraction: using Twisters with different types of phases can provide a more complete extraction and improved analyte recovery. Twister could be applied in GC and LC full automated analytical sessions by the Gerstel Multi-Purpose Sampler MPS 2. In the Twister applications, this autosampler could use a dedicated thermal desorption unit or system (TDU2 and TDS2) for GC analysis; involatile, polar, or thermally labile compounds can be extracted from the Twister with a proper solvent, using Twister Back Extraction (TBE) for subsequent determination by LC/MS [64]. In 2019, an SBSE was implemented with Ice Concentration Linked with Extractive Stirrer (ICECLES) to couple the benefits of SBSE and freeze distillation [65]. This combination led to a technique that preconcentrates and separates target compounds from matrix interferents at the same time. This technique does not need organic solvent if we use TD or only a small volume of organic solvent if we use backextracting, and it allows ultra-trace detection of a wide range of compounds in a liquid medium [66]. Coupling PAL system with design specific thermal units that provide both heating and cooling within the same block (−80°C to +250°C with a ±0.1°C), commercialized by Mècour (Grooveland, US), ICECLES can be completely automated. This company provides a full range of thermal units that accommodate media/reagent bottles or reservoirs to support various applications, integrable with stir plates and shakers required by ICECLES technology.

The MonoTrap-Monolithic Material Sorptive Extraction, based on Merck DGaA monolithic technology (GL Science Inc., Tokyo, Japan), is a sampler with a completely new design [67]. It has a porous silica monolithic hybrid surface, which extends the surface area, and contains activated carbon, graphite carbon, PDMS, and C18 functional group, which provides vast adsorption capacity. The sorbent phases enable rapid extraction by adsorption, complete desorption of the target compounds with a small amount of solvent, and they do not need a preconditioning step. The MonoTrap offers two different chemistries of adsorption: the first one is like silica gel, more appropriate for adsorption of nonpolar analytes, while the other one is like silica modified with activated carbon, as an enhancer of adsorption area to help the retention of more polar compounds. Both forms of monolithic material are functionalized with C18 groups, and they are offered in rod and disk designs, reusable numerous times after flushing. The monolithic-coated devices can be utilized for sampling highly diluted liquids and volatile chemicals prior to solvent or thermal desorption and GC or LC analysis [68, 69]. The MonoTrap analytical GC session can be fully automatized with the Multimode inlet-Optic 4 and the autosampler tool for liner change and desorption, LINEX. This instrumental setting used customized glass tubes for thermal desorption, the MonoTrap TD Liner for OPTIC/LINEX, to insert, store, and automatically desorb the sampled MonoTrap [70].

One of the newest nonexhaustive METs, the coated-blade-spray (CBS), has been developed and commercialized by RESTEK. CBS is a sword-like stainless steel sheet with an ultra-thin adsorbent phase (as hydrophilic-lipophilic balanced, HLB) that permits rapid extraction from complex medium, and it directly allows, once the coated area is wetted with a small quantity of solvent and a high-potential is applied to the noncoated area, the ESI ionization and the coupling with LC systems [71]. This open bed-SPE device is still developing, but its main features are already visible: minimization of matrix effect and ionization suppression, robustness and versatility for matrixes with different shapes, viscosities and stiffness, and easily coupling with analytical instrumentation, both LC-MS or directly in MS interface. Ionization interfaces and a completely commercialized solution are under development. Nowadays, CBS could fit with a new injection port for MS instruments to overcome the chromatographic separation and directly goes in the analyzer: the Touch Express Open Port Sampling Interface (OPSI) (Advion, New York, US), developed by Van Berkel and Kertesz. This tool allows direct assays from sample preparation tips and SPME fibers, and it could be applied to screening applications for drug research, food safety, environmental, and forensics [72].

Another novel nonexhaustive MET is the TF-SPME (JP Scientific Ltd. Waterloo, Ontario, Canada), available since 2019 from Gerstel GmbH. The TF-SPME is a sheet of 20 mm × 4.8 mm of carbon mesh impregnated with a sorptive phase, and it can be applied in headspace or immersion extraction. Liquids are most often extracted by immersing the TF-SPME device with a stir bar to agitate the liquid, while solids are extracted in headspace mode in an agitator. The available TF-SPME phases employ PDMS loaded with either carboxen (CAR), divinylbenzene (DVB), or HLB particles [73]. The geometry of TF-SPME devices enhances the sampling rate through its thin extraction phase and large surface area, providing a high surface-area-to-volume ratio. The sampling rate helps reduce the time needed to reach equilibrium while still increasing the capacity of the extraction device [74]. The planar geometry of the TF-SPME makes it compliant to on-site environmental sampling, as well as direct sampling of sample surfaces or skins [75]. To simplify the extraction, the TF-SPME can be coupled with SBSE and solvent back extraction, also achieving improvements in sensitivity and recovery of volatile compounds. The combined extraction technique is usually performed on liquids, where stirring of the sample is obtained thanks to the Twister, while the TF-SPME device is immersed in the sample [76]. The two devices can then be conveniently desorbed together in a single TD tube. The combination of Twister and TF-SPME was found to yield the highest responses for a large group of volatile compounds covering a wide range of polarity (log K_ow_ from -0.26 to 4.83) [77]. Using Gerstel MPS autosampler or Markes Centri, automated extraction can be achieved before GC analysis. However, once the TF is enriched with the target analyte, it must be transferred in a liner or in a metal desorption tube by hand to be analyzed by GC, through thermal desorption [78]. One possible application for high-throughput processing of aqueous samples to LC analysis is the Brush configuration, currently under product development through Supelco. It is composed of 96 TF devices set in a 96-well plate to carry out the extraction from the samples, which are subsequently moved to another 96-well plate with an extraction solvent to desorb the devices before the LC injection. This offline samples preparation can be accomplished with a manual workstation or via robotic ones that provide automation of all preconditioning, extraction, washing, and desorption steps [79]. Both solutions are available through PAS Technologies (Magdala, Germany).

Several efforts were made to develop a tool with the sorbent coating-packing inside of a needle or a glass shell with related higher extraction speed, greater capacity, and stability, to overcome the main drawbacks of SPME, as flexibility of surface area, film thickness, robustness, and durability of the external coating. The first attempt to develop this new kind of tool was made in 1970 by Cronin [80] with glass PLOT GC capillary columns for quantitative sample trapping. The first proposal for the inside needle capillary adsorption trap (INCAT) solution was by Fowler in 1979 [81]. In 1986, Burger and Munro [82] used fused-silica capillary traps with stainless steel tubes for fast thermal desorption by direct current heating. In 1997, Tuan et al. [83] showed the application of capillary traps coupled with a portable micro-GC system. The modern in-tube (IT) SPME with an open tubular fused-silica capillary column was designed by Eisert and Pawliszyn [84] for application with LC-MS, as an alternative to fiber SPME, because it could scarcely endure aggressive LC solvents. Later, Kataoka [85] and Globig and Weickhardt [86] provided an overview of robotized sample treatment by use IT-SPME and its applications for the analysis of polar and thermolabile chemicals in environmental, clinical, and food fields. In 2001, Pawliszyn's group proposed the Needle Trap MicroExtraction (NTME) (PAS Technology, Magdala, Germany) for particle sampling from the air, and it acted as a trap for particles rather than for volatile organic compounds (VOCs). In the NTME technique, commercial sorbents such as PDMS- DVB, Carboxen, Carbopack X, Tenax TA, and polymer-based beads are used to sample organic compounds from different media. Recently, Mieth et al. [87] showed that a combination of different sorbents into the same needle trap could enhance the extraction performance, while Maleki et al. [88] customized the NTME with nanoporous silica sorbents, showing the possible wide range of applications for this technique.

#### 2.2.2. Exhaustive Microextraction Techniques

Based on the NTME and IT extraction, several commercially available exhaustive devices were developed. However, the first IT device for headspace analysis was an exception, because it can be considered as a nonexhaustive technique: it was released by Chromtech (Idstein, Germany) in 2000, and it is commercialized as SPDE device, known as Magic Needle (SPDE-The Magic Needle). This tool comprises stainless steel needles (8 cm) coated with a film of sorbent material, applied in variable thicknesses, and 10% activated carbon [89]. The volume of the stationary phase of the SPDE needle is approximately 5.99 *μ*L higher than a 100 *μ*m PDMS SPME fiber (0.94 *μ*L), allowing short time and high-efficiency extraction. Generally, the Magic Needle is used with a gas-tight 2.5 mL syringe, and all sample preparation steps are fully automatized with autosampler, as PAL CTC: the sample, liquid or headspace, is drawn up into the device, thus forcing the analytes via the sorbent phase. After adsorption, the sample is thermally desorbed and analyzed by GC, using an autosampler to execute these steps. Because of the exhaustive nature of the device, additional care must be taken to ensure that no breakthrough occurs during extraction. A similar design device, the NeedlEX, was introduced on the market by Shinwa Chemical Industries Ltd (Kyoto, Japan) to sample the air with aspirating pump or manual gas-tight syringe to analyze alcohols, organic solvent, amine, and fatty acids [90]. This device does not require additional equipment for the desorption of samples, which is performed directly by GC injector, and can be used repeatedly (25 to 30 times), by conditioning it in the GC injection ports for 3 minutes after each analysis, allowing automation of analytical session using three-axis autosamplers.

One of the most used IT-MET was developed as a beta test version from BGB Analytik (Adliswil, Switzerland) in 2008 [23], and now, it is commercially available as ITEX-In-Tube Extraction by CTC Analytics. This solution was developed for dynamic HS, coupled with thermal desorption and GC determinations [91, 92]. ITEX is a syringe-based headspace enrichment technique robust, easy to use and, thanks to the sorbent trap, capable of achieving sensitivity at ppt. It is a gas-tight syringe with a micro trap with adsorbent material positioned inside the syringe needle to concentrate the target compounds from the sample's headspace. The trap could be filled with different material, as Tenax, Carbopack, Carbosieve, and Molecular sieve. The chance to carry out repeated strokes of the syringe from the headspace allows for a scalable sensitivity level, making this approach versatile towards low and high concentration samples. The device, after the enrichment step, is directly thermally desorbed in the GC injector, releasing the compounds into the inlet, and transferring the sample in a very narrow band to preserve the column efficiency [93]. Automation with ITEX is achievable with a three-axis autosampler, as PAL CTC or TriPlu RSH Autosampler (Thermo Scientific, Waltham, US), that also purchases a series of support tools to optimize its workflow, as PAL RTC (Robotic Tool Change), which can switch from ITEX to conventional syringe without human intervention.

Entech Instruments (Simi Valley, US) proposes an exhaustive MET, the Sorbent Pens, compatible with GC system. The sorbent pen uses the Vacuum-Assisted Sorbent Extraction technique (VASE), combining the advantages of both SBSE and vacuum HS-SPME, and coupling the features of SPME and classical adsorbent traps. They are packed generally with a larger quantity of extraction material than SBSE (10 times) and SPME (500 times). They are proposed in three versions: heads space, diffusive for long term environmental monitoring, and active for air sampling from 5 minutes to 8 hours. Sorbent Pen can be directly desorbed onto the head of a GC column, eliminating losses related with TD traps (like additional traps to focalize the analytes and lengthy transfer lines) [94]. They are extremely robust, and they allow sampling in the field and in the laboratory, either actively or passively or under a vacuum [95]. Sorbent Pen can face the requirements of different applications, as organic compounds extraction from a wide variety of different media, as wastewater, breath, or beverages. One of the main features of this device is the opportunity to use it under vacuum, overcoming in extraction efficiency SPME, Dynamic Headspace, and other extraction techniques that work at atmospheric pressure, limited slower diffusion rates [96]. Sorbent Pens can carry out offline sample enrichment from a GC system, enabling the simultaneous extraction of multiple samples. Sorbent Pen can also be easily applied to full automation thanks to the affordably Sorbent Pen Desorption Unit and the Entech's new Sample Preparation Rail, which allow the analysis of up to 120 preextracted devices using four removable 30-position racks [94].

A totally different design was used for the exhaustive Microextraction by Packed Sorbent (MEPS) system, patented and developed by SGE Analytical Science (Trajan Scientific and Medical, Ringwood Victoria, Australia). The MEPS couples extraction, preconcentration, and clean-up in a single solution. It is an SPE miniaturized device composed of a standard syringe adapted to host the BIN-2 mg circa of the extraction sorbent, packed as a plug between the needle and the barrel. The BIN has a mean particle size of 45 mm and can be packed with any absorption material, like silica-based, or new molecular imprinted polymers (MIPs) and restricted access material (RAM) [97]. MEPS could be managed manually or in fully robotized online systems joint with autosamplers compatible for a GC analysis or LC-elution [98]. A peculiar, automated device suitable for MEPS is the eVol XR, a digital analytical syringe that can work in full automation and perform high precision liquid handling procedures [99]. More conventional autosamplers that can manage MEPS are three-axis, as CT PAL, and the two-axis autosampler by HTA, the HT4000A, which can perform all the MEPS sample preparation and injection steps automatically.

Another exhaustive MET recently developed is the *μ*SPEed cartridge, commercialized in 2014 by EPREP PTY LTD (Oakleigh, Australia). It contains a micro one-way valve, patented in 2015 by E.F. Dawes, P.A. Dawes, R. Cerra, and A. Minett [100]. This MET is all contained in a unique cartridge, without the need for additional fittings or tubes. The peculiar valve allows drawing the sample through the syringe, eluding transit via the sorbent bed by pulling the plunger. Small particles can be used, offering a higher surface area, and allowing a more efficient separation, increasing the efficiency of the extraction step [101]. *μ*SPEed cartridges are reusable according to the used matrix and the working procedure. The *μ*SPEed cartridges are fully managed by eVol syringe and the new eXact Digital Syringe Driver (EPREP), which can cope with bigger back-pressures than the eVol. Such feature results are extremely helpful with complex media, such as biological fluids, that often lead to clogging. In addition, the EPREP Sample Preparation workstation allows the offline automation of *μ*SPEed cartridges, enabling to set sequential steps and to prepare samples away from analytical instrumentation and directly into a wide range of autosampler racks and then transfer them to the analytical instrument, as a chromatographic analyzer, reducing complexity in sample processes [102].

A particular different approach is represented by the SniffProbe (Aviv Analytical, Hod Hasharon, Israel), which is a synchronous pump that flows the air at a constant and stable pumping speed through 15 mm short pieces of standard 0.53 mm I.D. capillary or PLOT column, used as an exhaustive solid-phase microextraction trap for sampling air born, headspace, aroma, or air pollution. The SniffProbe traps commercially available are DB-5 ms with 1.5 *μ*m film, PoraBOND, CarbonPLOT, Micro SPE-Silicone, Mounted-Silicone, and MicroSPE-carbon. After the sampling, the short sections of the column are placed inside a pyrex vial (3 mm O.D., 10 mm length) with a 0.5 mm hole at its bottom or tailored glass microvial and introduced into a customized GC injector port for thermal desorption [103]. This injection port, known as ChromatoProbe, allows the direct introduction of the sorbent probe in the GC or GC/MS system and Aviv Analytical designed it, licensed to and available from 1997 by Varian and now available also by Agilent, under the name Thermal Separation Probe (TSP), by FLIR as PSI Probe, and by GL Science under the name DMI. SniffProbe needs the ChromatoProbe or similar, but it works well with standard Split/Splitless injectors and does not require a PTV injector for sensitive determination, thanks to the concentration step that could carry multiple loading cycles of the air sample on the extraction column. Offline automation of an analytical session of sampled column probes is achievable, using autosampler, as CTC PAL or similar, coupled to GC analyzers [104].

### 2.3. Robotics Automation

The automation of sample preparation can be done on the whole process or only on specific operations. Robotic autosamplers could carry out all the sample preparation steps, ranging from dilutions to derivatizations. In chromatography, automated, unattended sample treatment could increase laboratory throughput, diminish costs, offer results of higher precision than manual-operated tools, maximize work efficiency and data consistency/accuracy, and make any procedure applicable for routine analysis [105].

Nowadays, robotic autosamplers can be classified as cartesian, cylindrical, polar, and anthropomorphic. These configurations allow 3 degrees of freedom, and they can be easily coupled with analytical instruments to carry out the preanalytical phases online or offline. The transfer between automated segments, such as autosampler and GC or LC-MS analyzers, can be done online, without human intervention, or offline, requiring human intervention. The online systems, especially the cartesian ones, could be easily integrated on LC and GC systems thanks to tailored accessory instruments for sample clean-up, extraction, and injection; they can carry out a fully automated analytic session (Table 3). These devices could face the different needs of analytical methods and work without operators, simplifying a method development, improving the throughput of routine analysis, and delivering accurate results [106]. Instead, the offline robotic autosamplers require an operator to complete the analytical process (e.g., transfer of the prepared sample on the instrument for the analysis, decapping of the samples or injection). An offline autosampler may attend to prepare a vast number of samples without human intervention, carrying out a broad kind of operations (such as dispensing or weighting) (Table 3). Still, the intervention of an operator is required to proceed with the analyses. The automated change of device allows 24/7 operation with no operator, also for multistep process, and thus increases the output of labs. Simultaneously, since all operations necessitate less human involvement, process safety is optimized, thanks to shifting repetitive or dangerous manual jobs to a robotized system.

The first online autosampler was designed by CTC Analytics AG, which in 1986 released the A200S, the first GC liquid three-axis autosampler, evolved to the HTX PAL in 2003. Between 2012 and 2014, CTC Analytics commercialized the PAL system, as RTC, RSI, and LSI, becoming a reference point for analytical automation coupled with chromatographic systems. SPME solutions than can be managed by online modules, all of which are based on CTC Analytics' instruments, are nowadays produced as Value Added Reseller (VAR) or Original Equipment Manufacturer (OEM) by Shimadzu, Agilent Technologies, Thermo Scientific, Chromtech Analytical Instruments (Bad Camberg, Germany), Leap Technology Inc. (Trajan Scientific and Medical, Ringwood Victoria, Australia), Gerstel GmbH and Co. KG, Anatune Ltd. (Cambridge, United Kingdom), Da Vinci Laboratory Solution B.V. (Rotterdam, The Netherlands), JSB International (Eindhoven, Netherlands), SepSolve Analytical (Peterborough, UK), Markes International Inc., Entech Instruments, and Axel Semrau (Sprockhövel, Germany). Different autosamplers have been recently released, as FLEX (EST Analytical, Fairfield, US), ROBOKROM (KONIK Group, Barcelona, Spain), Primariz (Moduvision Technologies, Vlissingen, Netherlands), and CONCEPT MIS (PAS Technology). Between these, HTA has designed the HT2800 T, which is a new-concept two-axis sample preparation system more versatile than the older two-axis. The online autosamplers could be equipped with everything that could be necessary to entirely run SPME techniques, such as tool changer, vortex, centrifugation, balance, trays (allowing different sizes and temperatures), and barcode readers [107]. Frequently, new devices are released on the market to optimize the complete automation of the analytical process. Recently, Brechbuehler AG (Schlieren, Swiss) proposed the Grabber D885 (designed for CTC PAL and compatible with GC and LC techniques), a tool to overcome the problem of transferring nonmagnetic objects during the online automated sample preparation. This new device is equipped with an automated caliper capable of delivering sample tubes or spectroscopy cuvettes through any preparation steps. Thus, the online automation of sample preparation fulfils the requirements for analyzing many samples that need to be analyzed in some cases with high frequency [108]. The requirements to obtain high-throughput lab sessions drove to the development of dedicated systems for these METs, as automatic tool change -ATC (Markes Int. Inc) or robotic tool change-RTC (CTC Analytics) as Multi Fiber eXchange (MFX) (Chromline) for SPME fibers [109], Baker module (Chromtech Analytical Instruments) for large volume headspace extraction, and Thermal Desorption Unit tray (Gerstel) for Twister and MonoTrap, allowing online sampling and injection. Furthermore, to better manage the analytical process, both the analytical and preanalytical phases, numerous software devices are available to control the instruments remotely: Axel Semrau presented the Chronos software for analytical system controls (control all generation of PAL and compatible with tailored LIMS), while Gerstel GmbH introduced the Maestro system. In addition, Leap Technology offered the Workflow Notifier, compatible with Chronos software, that will alert by e-mail or text when an instrument needs an intervention, as when a sample list is completed, or an error has occurred.

**Figure 2:**
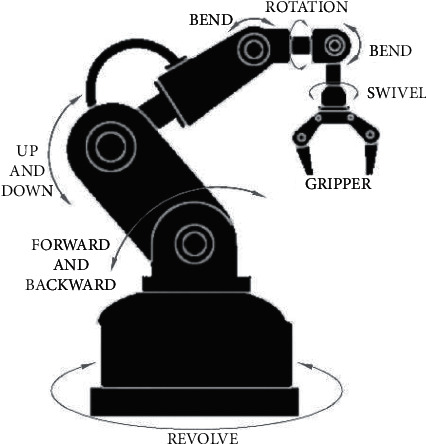
Example of the robotic arm with revolute joints.

Concerning the offline sample preparation systems that allow the use of modern METs, great prominence has been given to robotic anthropomorphic devices in recent years [105]. Thanks to highly developed research, these tools have already been established in analytical applications: robotic harms perform both transport and active manipulation tasks to ensure human-like operations and enabling the use of manual laboratory devices and equipment [110]. Zymate (Zymark Corp, Hopkinton, US) is one of the most used cylindrical coordinated robot arms with interchangeable hands and various workstations. Recently, other anthropomorphic robots with revolute joints have been released into the market, as ChromBot (Chromtech Analytical Instruments, Bad Camberg, Germany), a self-orientating robot with a load capacity of up to 500 grams, CHRONECT Quantos (developed in a cooperation of Axel Semrau with Mettler-Toledo, Jüke Systemtechnik, and Central Innovation Program for Medium-Sized Businesses) to dose powder samples, Andrew system (Andrew Alliance S.A., Vernier, Switzerland), a robotized polar tool to handle liquids, that uses volumetric pipettes, or Thermo Scientific F5, a anthropomorphic revolute 6 axes developed for laboratory automation with ±0.02 mm repeatability at full and 5 kg payload. These new robotic devices could carry out several activities, including all the movements required to use the most recent METs, also thanks to the flexibility obtained through the revolute joints (Figure 2). Moreover, other companies have developed modular, customizable laboratory robots that could be implemented with microextraction tools, as Sirius Automation (Illinois, US) with the Tasker series or Tecan (Buffalo Grove, Swiss) with Fluent.

The last frontier is represented by portable in vivo multisample desorption devices that allow the easy automated extraction of the exposure phase from multiple devices. The first high-throughput 96-well SPME-LC application used a pin-tool replicator to manage 96 pins with PDSM fibers fitted in them. After that, many applications of 96-fiber SPME in various geometry were proposed, using a multifiber system and Concept 96-autosampler (PAS Technologies) [111]. Innovations of high-throughput robotized SPME solutions have considerably reduced costs and time for analyses and increased their precisions as well. A future upgrade of these kinds of devices would be the 384- and 1536-well-plate designs to further increase sample throughput.

The automatization of a laboratory could require an important economic effort, but it is usually recovered from the reduction of the working force and the large number of processable samples. In this perspective, software devices compatible with sample automation systems have been released on the market to implement their application in clinical and research. Remarkably, chemometric and predictive software devices have been proposed to optimize the workflow, and the development of sample preparation methods, DryLab software (Molnar Institute for applied chromatography, Berlin, Germany), Chromcopesx (SepSolve Analytical), proEZGC (Restek), and Chromatogram Modeler (SGE), allows the prediction of chromatograms considering variable experimental settings, fast peak identification, easy-to-use deconvolution, and quantitation. This software results in a reduction of the number of analyses and costs that are needed to achieve the optimum instrumental conditions, according to analytical needs.

## 3. Conclusions

As this review has shown, the role of METs has become more relevant in all analytical fields, thanks to its compliance with green analytical requirements. The METs are currently increasingly developed, because their potential applications are vast. This increasing interest in METs led to a rapid expansion of the solutions to carry out miniaturized and automatized sample preparation. In this wide scenario, this study gives an overview of the commercially available METs and their solutions for automated sample managing to offer a valuable tool to customize an analytical activity as robust, efficient, reproducible, environmentally friendly, and economic as possible.

## Figures and Tables

**Table 1:** Nonexhaustive METs and their main features.

Extraction device	Name *Producer*	Design characteristics	Extraction phase			GC injection port	Automation
			Area (mm^2^)	Volume (*μ*L)	Sorbent		
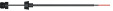 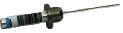	SPME *Supelco-Restek-Pas Tech.*	Conventional 100 *μ*m × 10 mm coating–0.7 mm o.d.	40	0.9	PDMS-PA-CAR/PDMS-PEG-DVB/CAR/PDMS-PDMS/DVB	Conventional (liner 0.75 mm i.d.)	Online Offline

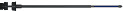 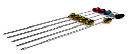	SPME-arrow *Restek Corp.*	1.1–1.5 needle o.d.	44 to 62.8	3.8 to 11.8	PDMS-PA-carbon WR/PDMS-PDMS/DVB-DVB/Carbon WR-PDMS	Conventional (liner 2.0 mm i.d.)	Online Offline

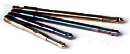	Hi-Sorb *Markes Int.*	Standard (8 cm) or Short (4 cm) length	65	65	PDMS-PDMS/DVB-PDMS/CWR-DVB/CWR/PDMS	HiSorb extraction module	Online Offline

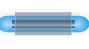 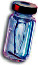	Twister *Gerstel GmbH*	10–20 mm length	—	63 to 126	PDMS-PDMS/EG	TDU or TDS	Online Offline

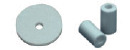	Monotrap *GL Science Inc*	2.9–10 mm diameter	45 to 160	—	AC/C18-C18-GRAPHITE/C18-GRAPHITE/PDMS	Optic-4	Online Offline

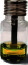 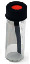	TF-SPME *Gerstel GmbH*	40 × 4.85 × 0.04 mm	198	200	PDMS-PDMS/DVB-PDMS/CAR-PDMS/HLB	TDU	Online Offline

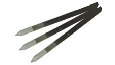	CBS *Restek Corp.*	42 mm length	30	—	PAN/HLB	OPSI	Online

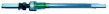	NTME *Pas Tech.*	Gauge 22 or 23 Length of 50–80 mm	3.0	—	Tenax- PDMS-DVB-carbopack-carboxen-carbosieve	Conventional (liner split/splitless)	Online Offline

PDMS: polydimethylsiloxane; PA: polyacrylate; CAR: carboxen; PEG: polyethylene glycol; DVB: divinylbenzene; Carbon WR or CWR: carbon wide range; AC: activated carbon; HLB: hydrophilic-lipophilic balanced; PAN: polyacrylonitrile; TDU: thermal desorption unit; TDS: thermal desorption system; OPSI: open port sampling interface.

**Table 2:** Exhaustive METs and their main features.

Extraction device	Name *Producer*	Design characteristics	Extraction phase			GC injection port	Automation
			Area (mm^2^)	Volume (*μ*L)	Sorbent		
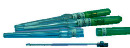	Needlex *Shinwa Chem Ind. LTD*	i.d. 0.5 mm-o.d. 0.7 mm-length 85 mm	3.0	—	Tenax- PDMS-DVB-carbopack-carboxen	Conventional (liner split/splitless)	Online Offline

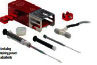	ITEX *CTC Anal. AG*	ITEX syringe 1300 *μ*l	3.0	—	Tenax-carbopack-carbosieve-carboxen	Conventional (liner split/splitless)	Online

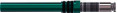 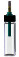	Sorbent Pen *Entech Inst.*	6.1 mm o.d. Length 88.9 mm	—	10	Tenax-tenax/carbopack-PDMS/tenax-PDMS/tenax/carbopack-carboxen-carbopack-PDMS/tenax-tenax/carbopack	SPDU-5800	Online Offline

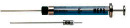	MEPS *SGE Anal. Sci.*	Syringe 100–250 *μ*L removable needle	<1.0	—	C18–C2-silica-C8-C8/SCX-SAX	Conventional (liner split/splitless)	Online Offline

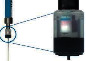	*μ*SPEed *ePREP*	Length <1 cm, 3 *μ*m sorbent particle size	<1.0	—	C18-WAX-PFAS-PS/DVB-silica-PS/DVB/Phenyl-SAX_PS/DVB-SCX_PS/DVB-cxyl	Conventional (liner split/splitless)	Online

i.d.: internal diameter; o.d.: outer diameter; PDMS: polydimethylsiloxane; DVB: divinylbenzene; AC: activated carbon; PEG: polyethylene glycol; PS: polystyrene; SCX: strong cation exchange; SAX: strong anion exchange; WAX: weak anion exchange; SPDU: sorbent pen desorption unit.

**Table 3:** Automated autosampler for sample preparation (online or offline) and their main characteristic and tools.

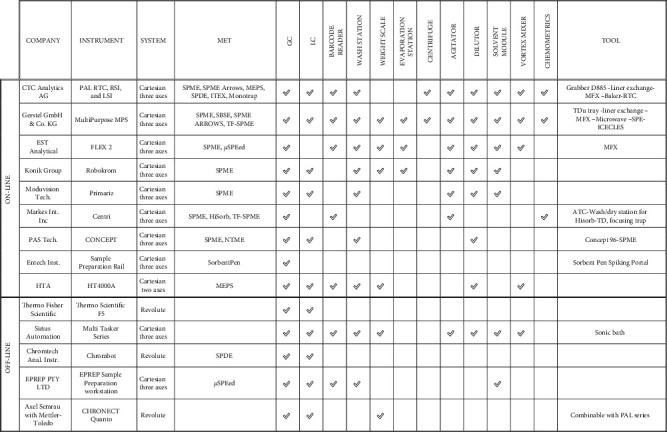

## Data Availability

No data were used to support this study.
